# Single-trial linear correlation analysis: application to characterization of stimulus modality effects

**DOI:** 10.3389/fncom.2013.00015

**Published:** 2013-03-18

**Authors:** Christoforos Christoforou, Fofi Constantinidou, Panayiota Shoshilou, Panagiotis G. Simos

**Affiliations:** ^1^Center for Applied Neuroscience, University of CyprusNicosia, Cyprus; ^2^Research and Development Division, R.K.I Leaders Ltd.Larnaca, Cyprus; ^3^Department of Psychology, University of CyprusNicosia, Cyprus; ^4^Department of Psychology, University of CreteRethymnon, Greece

**Keywords:** EEG, single-trial analysis, neuroimaging, correlated components, machine learning

## Abstract

A key objective in systems and cognitive neuroscience is to establish associations between behavioral measures and concurrent neuronal activity. Single-trial analysis has been proposed as a novel method for characterizing such correlates by first extracting neural components that maximally discriminate trials on a categorical variable, (e.g., hard vs. easy, correct vs. incorrect etc.), and then correlate those components to a continues dependent variable of interest, e.g., reaction time, difficulty Index, etc. However, often times in experiment design it is difficult to either define meaningful categorical variables, or to record enough trials for the method to extract the discriminant components. Experiments designed for the study of the effects of stimulus presentation modality in working memory provide such a scenario, as will be exemplified. In this paper, we proposed a new approach to single-trial analysis in which we directly extract neural activity that maximally correlates to single-trial manual response times; eliminating the need to define an arbitrary categorical variable. We demonstrate our method on real electroencephalography (EEG) data recordings from the study of stimulus presentation modality effect (SPME).

## 1. Introduction

A common challenge in the study of cognitive systems is to identify neural correlates of the different cognitive functions. In human subjects, the underlying neural activity is often measured using multi-channel electroencephalography (EEG), while the cognitive function is characterized behaviorally; typically by recording subjects' responses to external stimuli during performance of a task designed to elicit the cognitive function under study. A number of such paradigms have been proposed in the literature for the study of perceptual binding (Ehm and Bach, 2011), memory workload (Murata, 2005), attention (Tiitinen et al., 1993), arousal (Strber et al., 2000), object recognition (Basar-Eroglu et al., 2000), language perception (Eulitz et al., 1996), and decision making (Philiastides et al., 2006), among others. The challenge is then to identify components in the EEG signal that correlate with the behavioral variables.

Traditionally, identifying such neural correlates involves selecting specific channels (or channel groups), time windows and/or frequency bands and defining pertinent signal attribute [e.g., the amplitude or latency of a peak in the event related potential (ERP), or the magnitude of instantaneous power in the ongoing electroencephalogram (EEG)]. To increase the signal-to-noise ratio of these attributes, they are often obtained by averaging across multiple-trials. These mean values are then correlated with a behavioral/psychological parameter of interest (individual subject characteristic or performance measure). These methods require *a priori* decisions regarding which recording channels, time points and frequency bands are more likely to capture the neuronal activity of interest; which is often not the case, in particular in novel paradigms. In addition, traditional analysis methods are limited to identifying signal parameter modulation across subjects, whereas in typical experiments it is the instantaneous variations in behavioral and electrophysiological parameters that best capture the psychological phenomenon under investigation (e.g., the recognition of a particular stimulus).

Single-trial discriminant analysis has been proposed as a novel method for identifying components in ERP/EEG signals that could, in turn, be correlated with behavioral parameters indicative of a cognitive function (Vavatzanidis et al., 2010). Traditionally in this method, ERP/EEG epochs are initially assigned to one of two experimental conditions, and the single-trial analysis seeks to find projections of the multivariate ERP/EEG signal, within a short time window, that maximally discriminate between these two conditions. The resulting components capture neural activity associated with condition one, while mathematically minimizing activity associated with the other condition. In addition, single-trial discriminant analysis provides a more comprehensive estimate of these components by optimally integrating spatial information from multiple electrodes, thus increasing the signal-to-noise ratio. The amplitude of the resulting components captures more of the signal of interest and less of the noise in the signal than any of the individual channels alone. Finally, the amplitude of extracted components on a single-trial basis can be correlated to relevant behavioral variable (such as mean reaction time or error rate across subjects). The effectiveness of single-trial discriminant analysis depends on the availability of one or more categorical variables that split the trials into meaningful conditions. In addition, the experimental paradigm should allow for a sufficient number of trials to be collected, in approximately equal numbers for both conditions. However, often times the design of the experiment is such that it is either not possible to define a meaningful categorical variable, or one of the conditions has insufficient trials. A common technique to alleviate the first limitation has been to convert the continuous variable, which measures the behavioral effect of interest, into a categorical one by setting a criterion (see for instance, Vavatzanidis et al., 2010). However, this approach is not void of serious validity issues. For instance, the choice of the criterion for discretizing a continuous variable is often arbitrary and may not bear the intended relevance with the cognitive function under study and/or the underlying neurophysiological operations. Moreover, the number of trials available for analysis may be significantly reduced, since a large number of trials that fall around the boundary of the threshold are typically discarded.

In this paper, we propose a novel approach to single-trial analysis in which extraction of relevant EEG components relies from the outset on their measured association with the continuous behavioral variable of interest (here, reaction time), eliminating the need to define an arbitrary categorical variable. This method bears all the advantages of single-trial discriminant analysis and extents its applicability to paradigms where it is difficult to define neurophysiologically and/or cognitively meaningful categorical variables. In addition, this approach results in EEG components that optimally characterize the behavioral effect of interest (speed of processing and decision time) on a continuous scale. Here we demonstrate the effectiveness of the method in the analysis of previously unpublished data from a cross-modal verbal learning experiment, aiming to identify EEG components related to unimodal vs. cross-modal encoding of words. The paper is organized as follows: we first briefly review the single-trial discriminant analysis method in section 2.1 and then introduce our proposed method of single-trial linear correlation analysis in section 2.2. Next the issues of regularization, forward model estimation, and estimation of correlated components are presented in section 2.2. Details on the behavioral experiment data acquisition and preprocessing are outlined in section 3, followed by presentation of results from applying the method in order to characterize the stimulus modality effect (section 4).

## 2. Materials and methods

### 2.1 Single-trial discriminant analysis—brief overview

Various techniques and applications of single-trial discriminant analysis have been described in depth previously (Dyrholm et al., 2007; Parra et al., 2009; Christoforou et al., 2010; Sajda et al., 2010). In this section, we briefly review its key characteristics. Single-trial discriminant analysis seeks to identify a linear projection of multi-channel EEG signals, within a short time window, that maximally differentiates between trials from two behaviorally and/or cognitively distinct conditions. Let **x**_*n*_(*t*) ∈ ℝ^*D*^ correspond to the EEG activity recorded at time *t* and trial *n* from *D* sensors. A spatial weighting vector **w** ∈ ℝ^*D*^ is used to generate a one-dimensional projection *c*(*t*) ∈ ℝ of the *D* EEG channels,

(1) $$c(t)=w^{⊤}x(t)$$

The method requires each trial to be associated with one value of a binary, categorical variable *y*_*n*_ ∈ {−1, 1}. The method then estimates the vector **w** such that the values to *c*(*t*) maximally differentiate trials in the two classes [i.e., *y*_*n*_ = *f* (**w**^⊤^**x**_*n*_(*t*)), where the function *f*: ℝ → {−1, 1} is typically the logistic function]. A fundamental assumption in this approach is that the categorical variable *y* corresponds to meaningful modulation of underlying neuronal activity within each subject. If that is the case then the extracted electrophysiological components *c*(*t*) can be correlated with the continuous behavioral variable across subjects.

### 2.2 Single-trial correlation analysis

In this section we introduce our proposed single-trial correlation analysis method. Let **x**_*n*_(*t*) ∈ ℝ^*D*^, be the *D*−dimensional EEG data vector at time *t*, and *y*_*n*_ ∈ ℝ the behavioral response variable (i.e., subjects manual reaction times), at trial *n*. The elements of vector **x** could represent either ERP/EEG amplitudes, or the instantaneous power in selected frequency bands. Note that unlike single-trial discriminant analysis, the response variable here is continuous. Let **X**^*t*^ ∈ ℝ ^*D* × *N*^ be the data matrix with columns corresponding to the vectors of **x**_*n*_(*t*) for the *N* trials, and **y**_resp_ ∈ ℝ^*D*^ the behavioral response vector, as follows:

(2) $$X^{\left(t\right)}=[x_{1}(t),x_{2}(t),\ldots ,x_{N}(t)]$$

and

(3) $$y_{\text{resp}}={\left[y_{1},y_{2},\ldots ,y_{N}\right]}^{⊤}$$

The goal of the method is to identify a spatial weighting vector **w** ∈ ℝ^*D*^ such that the resulting EEG vector **y**_eeg_ = **X**^⊤^**w** maximally correlates with the behavioral response vector **y**_resp_. Formally, the optimization problem seeks to maximize the Pearson product moment correlation between **y**_eeg_ and **y**_resp_:

(4) $$\begin{array}{l} {\hat{w}}^{\left(t\right)}=\arg_{w}\max\frac{y_{\text{eeg}}^{⊤}y_{\text{resp}}}{∥y_{\text{eeg}}∥∥\;y_{\text{resp}}∥}\\ \;=\arg_{w}\max\frac{{\left(X^{⊤}w\right)}^{⊤}y_{\text{resp}}}{\sqrt{{\left(X^{⊤}w\right)}^{⊤}(X^{⊤}w)}\sqrt{y_{\text{resp}}^{⊤}y_{\text{resp}}}}\\ \;=\arg_{w}\max\frac{w^{⊤}Xy_{\text{resp}}}{\sqrt{w^{⊤}XX^{⊤}w}\sqrt{y_{\text{resp}}^{⊤}y_{\text{resp}}}}\\ \;=\arg_{w}\max\frac{w^{⊤}Xy_{\text{resp}}}{\sqrt{w^{⊤}R_{\text{xx}}w}\sqrt{y_{\text{resp}}^{⊤}y_{\text{resp}}}} \end{array}$$

where $R_{\text{xx}}=\frac{1}{N}XX^{⊤}$ is the estimated covariance matrix of the data. By taking the derivative of equation (4) with respect to **w**, setting it to zero and solving for **w**, we obtain the optimal solution for $\hat{w}$ by:

(5) $${\hat{w}}^{\left(t\right)}=R_{\text{xx}}^{-1}Xy_{\text{resp}}$$

Please note that superscript ^(*t*)^ on **X** has been omitted in Equations (4) and (5) for simplicity.

#### 2.2.1. Regularization

A common theme in optimization problems that involve highly-dimensional data is that of over fitting the model parameters due to limited sample size. To ensure good generalization performance for new data, regularization constraints are often introduced. Typically, these constrains apply prior knowledge on the particular type of data on certain properties of the solution. Thus, they favor solutions that comply with such prior expectations and penalize solutions that violate them. Common regularization constraints used in the literature are the L1- and L2-norms (Parra et al., 2008) favoring *sparse* and *smooth* solutions, respectively. In our case, we introduce two types of regularization constrains. First, we enforce a structure on the covariance matrix, and the response vector **y**_resp_ based on prior knowledge that relevant activity in the EEG signals changes slowly (compared to the actual signal sampling rate). Accordingly, temporally adjacent samples measure similar, relevant activity, within a short time window (Parra et al., 2008). This constraint is enforced by augmenting the data matrix **X** and the response vector **y**_resp_ as follows:

(6) $${\tilde{X}}^{t}=\left[X^{t},X^{t\;+\;1},\ldots ,X^{t\;+\;\Delta t}\right]$$

(7) $${\tilde{y}}_{\text{resp}}=1⊗{\left[y_{1},y_{2},\ldots ,y_{N}\right]}^{⊤}$$

where ⊗ here denotes the kroneker delta product between two vectors, Δ*t* ∈ ℤ_>1_ corresponds to the length of the short local window in samples and **1** corresponds to the Δ*t*-dimensional column vector of ones. Please note that the vector ỹ_resp_ has *N*Δ*t* dimensions. Also note that this definition of the data matrix, generates a more accurate estimate of the sample correlation matrix **R**_xx_, providing Δ*t*-fold more samples in the estimation, and therefore allows for a more accurate estimation of vector **w** (Equation 5). Further, the block structure defined on ỹ_resp_ enforces minimization of the correlation error within the entire window of length Δ*t* since all local samples will contribute to the joint fixed correlation.

Second, we introduced an *L*_2_-norm regularization prior on the parameter vector **w**. As a result the optimization problem in Equation (4) is finally expressed as:

(8) $${\hat{w}}^{t}=\arg_{w}\max\frac{w^{⊤}Xy_{\text{resp}}}{\sqrt{w^{⊤}R_{\text{xx}}w}\sqrt{y_{\text{resp}}^{⊤}y_{\text{resp}}}}+\frac{\lambda }{2}\;∥w{∥}^{2}$$

and the solution corresponds to:

(9) $${\hat{w}}^{t}={\left(R_{\text{xx}}+\lambda I\right)}^{-1}Xy_{\text{resp}}$$

where λ controls the influence of the regularization term on the solution and **I** is the identity matrix. The value of λ can be chosen through a cross-validation procedure on an independent sub-set of the data. The *L*_2_ prior controls the smoothness of the coefficients **w**, taking advantage of the prior expectation that neighboring electrodes measure similar activity. In essence, the two types of regularization enforce smoothness both in space (*through L*_2_ *regularization*) and in time (*through covariance structure*).

#### 2.2.2. Correlated components

Let **w**^(τ)^ be the optimal weighting vector obtained from Equation (8) using measurements at time sample τ (see section 2.2.4 for selecting τ). We can define the single-trial correlated component (SCC) *z*_*n*_ for *n*th trial as follows:

(10) $$z_{n}^{\tau }(t)=w^{(\tau )⊤}x_{n}(t),$$

and the component correlation trace (CCT) *c*(*t*) across all trials within a group as:

(11) $$c^{\tau }(t)=\frac{w^{(\tau )⊤}X^{\left(t\right)}y_{\text{resp}}}{\sqrt{w^{(\tau )⊤}XX^{⊤}w^{\left(\tau \right)}}\sqrt{y_{\text{resp}}^{⊤}y_{\text{resp}}}}$$

The SCCs of Equation (10) capture neural activity that maximally correlates with the behavioral variable and minimizes unrelated neural activity. Therefore, SCCs carry information relevant to the cognitive functions of interest at a higher signal-to-noise ratio. The amplitude of SCCs may thus serve as a measure of differences between groups, or between experimental conditions at the neural level. In addition, SCCs capture the temporal dynamics of the neural activity of interest, and can be used to characterize the latency and temporal modulation of this activity. Finally, SCCs from multiple-trials can be used to capture the evolution of different components of neurophysiological activity during engagement of a particular cognitive function in real time.

The CCT of Equation (11) shows how the strength of the association between the neural activity features, captured by the spatial component vector **w**, and reaction time varies over time (within the recorded epoch). Peaks in the CCT indicate the latencies at which a particular feature of neural activity becomes functionally relevant to the subject's decision and response to the stimulus.

#### 2.2.3. Forward model

Similar to the single-trial discriminant methods, our proposed method allows for recovering the “forward model” (Parra et al., 2008), which can be used to visualize the topographic distribution of the correlated components. The model is defined as:

(12) $$a_{\tau }=\frac{Xz^{\tau }(t)}{z^{\tau }{\left(t\right)}^{⊤}z^{\tau }(t)}$$

where vector **z**^τ^(*t*) =[*z*^τ^_1_(*t*), *z*^τ^_2_(*t*),…, *z*^τ^_*N*_(*t*)]. Typically, one is interested to recover the forward model on the time point the optimal windows is identified (i.e., where *t* = τ). The vector **a** captures the electrical coupling of the correlated component **z** which explains most of the activity in **X** that correlates with the response variable. The forward model can be visualized on the scalp surface and subsequently used as input to source localization algorithms in order to estimate the anatomical origin of these components.

#### 2.2.4. Determine τ, selecting significant time windows

In order to determine the temporal dynamics of the correlated components within the recorded epoch and determine the optimal window offset τ, we repeatedly trained our model on successive, partially overlapping time windows (of 60 ms duration in increments of 10 ms, starting from stimulus onset at 0–1000 ms post-stimulus). The performance of each correlated component at each window was indicated by the strength of the correlation coefficient between the resulting SCC amplitude (across trials) and the behavioral variable using a five-fold cross-validation procedure. Statistical significance of the correlation coefficients was established using a permutation test. In particular, we established a non-parametric distribution of correlation values (under the null hypothesis) by repeatedly (1000 times) assuming random assignment of the response variable across trials, training our model in a five-fold cross-validation procedure, and calculating the correlation between the resulting SCC amplitude and the behavioral variable. The false recovery rate method (FDR) was used to control for Type I error in determining statistical significance. The optimal window offset *tau* is selected among all window offsets that show significant correlation according to the procedure just described. In particular, the *tau* is selected as the offset for which correlation coefficients across time peaks (i.e., is a local maximum) along all statistically significant window offsets. The window selection procedure is run on an independent subset of the data.

## 3. Application

### 3.1 Stimulus presentation modality effect

The stimulus presentation modality effect (SPME) refers to differences in performance observed in various memory tasks due to the modality of the stimuli to be memorized. Constantinidou and Baker (2002) employed a multitrial verbal learning paradigm to investigate the effects of presentation modality (auditory, visual, or simultaneous auditory plus visual) with healthy and neurologically impaired younger and older adults. The goal of those studies was to determine which presentation modality could enhance learning and memory performance and therefore facilitate cognitive rehabilitation efforts. Results showed that the visual and the cross-modal presentation systematically yielded improved verbal learning and recall performance as compared to the auditory modality alone. Here, we sought to identify time-dependent components in single-trial, multi-channel EEG data obtained in response to the word stimuli during the encoding phase of this task. The single trial correlation analysis was applied in order to determine which EEG components (in the temporal and spatial domains) were closely related to the subject's response times on each trial and were therefore more likely to reflect cognitive demands of the task.

The principle underlying our proposed approach to EEG signal analysis is that neurophysiological activity recorded in the form of surface voltage fluctuations is produced by neuronal aggregates implicated in the brain mechanism (or circuit) responsible for a particular psychological function. The latter is actualized in the context of one or more relevant experimental tasks, typically requiring an overt response from the participant to indicate their decision regarding a stimulus attribute. In the present case, participants produced a manual response upon deciding whether a spoken or printed word was encountered previously in the context of the same experiment. The accuracy and particularly the speed of this behavioral response is typically considered to be an outcome index of the efficiency of the brain mechanism under study. As noted previously, neurophysiological activity recorded in real time during task performance may be probed for indices of the processing efficiency of that mechanism. It is surmised that extracted “components” of the recorded neurophysiological activity that correlate more strongly in real time (which also implies on a trial by trial basis) with reaction time, will be those more intimately implicated in the brain mechanism responsible for processing and responding to the experimental stimuli. Accordingly, the goal of the present study was two-fold: First, to develop an algorithm to identify task-relevant EEG components on a single-trial basis. Second, to validate this exploratory approach (establish that extracted EEG components are cognitively relevant). The latter goal was pursued by examining stimulus modality effects on each of the extracted components. Thus, in addition to the theoretical significance of identifying EEG components as indices of neurophysiological processes which are instrumentally linked to efficient task performance, our analytic approach represents a step toward establishing the validity of the method for future applications where the boundaries between task conditions may not be as clear cut. For instance, in the case where two experimental conditions are defined by stimulus type, but the actual mechanism engaged to process each stimulus is determined *ad hoc* by complex interactions between antecedent events and subject traits, that would be the case where subject characteristics influence the evaluation of particular stimuli by modulating situational expectations.

### 3.2 Experimental paradigm

#### 3.2.1. Subject

Data from seven healthy volunteers (aged 18–24 SD = 2.4 years) are reported here. All had normal or corrected to normal vision and reported no history of neurological disorder or learning disability. Informed consent was obtained from all participants in accordance with the guidelines and approval of the University of Cyprus Ethics Committee.

#### 3.2.2. Stimuli

A different set of 60 stimuli were prepared for each of three conditions (auditory, visual, audiovisual) consisting either of concrete and highly imageable object names in Greek (auditory and audiovisual conditions) or line drawing of objects (visual and audiovisual conditions). Fifteen stimuli from each list were identified as *targets*, and the other 45 as *foils* (15 foils per recognition block). In the auditory condition subjects listened to recordings of object names. In the visual condition they viewed line drawings of objects on a computer screen. In the audiovisual condition participants listened to the name of the depicted object the corresponding line drawing was presented. Moreover, individual foils were chosen for each of the three target lists so that they were similar semantically or phonetically to the target items.

#### 3.2.3. Experimental task

The experimental paradigm was modified from its original version, described in Constantinidou and Baker (2002) in order to facilitate EEG data recording, by replacing the free-recall test employed in the original paradigm with a recognition test permitting measurement of reaction times to individual stimuli during retrieval of previously encoded stimuli (targets). Modality conditions were administered in separate sessions in counterbalanced order. Each session is composed of five blocks, and each block includes a memorization task, and a recognition task. For the memorization task participants were presented with the target list items which were asked to try to memorize for subsequent recall. The memorization task was followed by a recognition task involving a set of 30 items (the 15 targets and 15 foils) and asked to press one key for targets and a second key for foils as fast as possible. Within each session all items were presented in the same modality, one at a time for 1500 ms. The experimental task was designed for an ongoing study that aims to identify neurophysiological correlates of the Stimulus Modality Effect. In this paper we use a section of the initially recorded data for the purpose of demonstrating the applicability of our method. In addition to the main experimental task, each subject participated in an eye-movement calibration task providing data for subsequent ocular artifact correction. During the eye-calibration, the subject was asked to first blink repeatedly for 10 s, and then to follow a cross on the screen moving first in a rightward and then in a downward direction (for 10 s each).

### 3.3. Data acquisition

EEG data were acquired continuously using a BioSemi Active Two system (BioSemi, Amsterdam, Netherlands) equipped with 128 EEG channels positioned according to the Biosemi extended version of the 10/20 international system. In addition, the Electrooculogram (EOG) was recorded from eight additional channels. Data where sampled at 512 Hz and impedances for all sensors were kept below 20 *k*Ω.

### 3.4. Data preprocessing

All signal processing was performed offline using MATLAB (Mathwords, Natick, MA, USA). A software-based 1.5 Hz high-pass filter was first used to remove DC drifts, followed by application of 50 and 100 Hz notch filters to minimize power-line interference.

#### 3.4.1. Ocular artifact removal

Data recorded during the calibration session where used to estimate spatial projections that capture ocular activity. In particular, three such components where estimated for three types of ocular artifacts (eye-blinks, left–right, and top–bottom saccades). Subsequently, activity captured by these components was removed from the EEG recordings using the method described in Parra et al. (2005), having first visually confirmed that each component bore the prototypical appearance of ocular artifacts.

#### 3.4.2. Epoching, re-referencing, artifact rejection

Following ocular artifact removal EEG data were re-referenced to the average-channel and then epoched between −700 and 1000 ms after stimulus onset. Then, the baseline amplitude from −300 to −100 ms was removed from each epoch. Subsequently, the trial auto reject method implemented in EEGlab (Delorme and Makeig, 2004) was used to identify and remove trials containing residual artifacts (method *pop_autorej* with default parameters). The data set available for EEG analysis (aggregating target and foil trials) consisted of 200–210 trials per subject.

#### 3.4.3. Spectral transformation

Power in different frequency bands has been widely explored as a measure of neural activity associated with task performance (Roach and Daniel, 2008). Hence, for our analysis we calculated the time/frequency decomposition of each channel and each trial. The time/frequency coefficient *F*(*c, t, f, n*) of channel *c*, time *t*, frequency *f*, and trial *n* is obtained by convolving the raw EEG signal with a complex morlet kernel of the form $\phi (t)=\alpha e^{2\pi ift}e^{{\left(-\frac{t}{\sqrt{2\sigma }}\right)}^{2}}$, where the parameter *f* corresponds to the kernel frequency, σ corresponds to the standard deviation of the Gaussian envelop, and alpha is α is a scale parameter. Note that the coefficients *F*(*c, t, f, n*) are complex-numbers. We can then define the instantaneous power for each frequency band, channel, time window and trial as:

(13) $$\bar{F}(c,t,f,n)=\;|F(c,t,f,n){|}^{2}$$

All subsequent analyses were performed on the instantaneous power tensor $\bar{F}(c,t,f,n)$, computed for each subject, rather than the raw EEG signals.

### 3.5. Datasets definition

To evaluate our method, behaviorally-relevant SCCs were sought in separate datasets according to standard EEG frequency bands as follows: ℱ_θ_ = (5–7 Hz), ℱ_α_ = (8–12 Hz), ℱ_β 1_ = (16–22 Hz), ℱ_β2_ = (23–30 Hz), and ℱ_γ_ = (31–40 Hz).

#### 3.5.1. Frequency datasets

For each frequency band {ℱ_θ_,ℱ_α_,ℱ_β 1_ ℱ_β 2_,ℱ_γ_}, and each modality condition *m* ∈ {*auditory, visual, audiovisual*}, the subject-specific data tensor was defined as follows:

(14) $$D_{s,m}^{\left(ℱ\right)}=\underset{f\in ℱ}{\max}{\bar{F}}_{s,m}(c,t,n,f)-M_{s,m}$$

Where ${\bar{F}}_{s,m}$ is the instantaneous power on all trials obtained from subject *s*, in modality condition *m* for both *target* and *foil* trials. Tensor **M**_*s,m*_ is the mean instantaneous power during stimuli encoding trials, of subject *s* and modality *m*. Then we can define the modality-specific data tensor **D**^(ℱ)^_*m*_ ∈ ℝ ^*D* × *T* × *N*^ as the aggregation of ${\bar{F}}_{s,m}$ across all subjects.

For a particular time-point *t* and window Δ*t* we can construct the data matrix ${\tilde{X}}^{\left(t\right)}$ and the response variable ỹ_resp_ of Equations (6) and (7), respectively, for each frequency band and modality condition, where the data matrix **X**^(*t*)^ in the equation now corresponds to the columns of tensor **D**^(ℱ)^_*m*_ across its third-way (i.e., across the dimensions representing the individual trials).

#### 3.5.2. Training set, cross-validation, test sets

For each frequency-modality dataset **D**^(ℱ)^_*m*_, we randomly assigned 80% (*n* ≈ 700) of the trials into a Training/Cross-validation dataset, denoted by Ḋ^(ℱ)^_*m*_, and the remaining trials (*n* ≈ 200) to an Test dataset denoted as ${\overset{..}{D}}_{m}^{\left(ℱ\right)}$. The first is used to identify the optimal time windows and cross-validate the performance of the extracted component. The independent test dataset is used to validate the predicted components.

## 4. Results

### 4.1. Behavioral results

A repeated measures ANOVA was used to examine the effects of condition (auditory, visual, and audiovisual), learning trial (1–5), and stimulus type (target vs. foil) on recognition RTs. Results showed the expected modality main effect, *F*_(2, 10)_ = 75.949, *p* = 0.0001, partial *η*^2^ = 0.938, power = 1.0. Pairwise Helmert contrasts showed that the auditory condition was associated with slower RTs than the visual and audiovisual conditions, *F*_(1, 11)_ = 161.784, *p* = 0.0001, partial *η*^2^ = 0.936, power = 1.0, which did not differ from each other, *p* > 0.5. Additionally, there was a significant linear trend of learning trials indicating that subjects became faster across the five trials, demonstrating a learning effect, *F*_(4, 44)_ = 8.960, *p* = 0.0001, partial *η*^2^ = 0.449, power = 0.998. Finally, participants showed faster reaction times to targets as compared to foils (Type main effect), indicating greater level of familiarity with the target items, *F*_(1, 11)_ = 10.292, *p* = 0.008, partial *η*^2^ = 0.483, power =0.831. Finally, there was a type by trials interaction effect, *F*_(4, 8)_ = 8.543, *p* = 0.005, partial *η*^2^ = 0.810, power = 0.951. In summary, this modified behavioral paradigm adapted for the EEG experiment yielded results consistent with our previous findings demonstrating a robust modality effect on average RTs.

### 4.2. EEG analysis results

For the EEG analysis results we report our finding on two, out of the three modalities, namely visual and audio-visual, and only for those frequency-band datasets for which our method was able to extract meaningful correlated components.

#### 4.2.1. Time window selection

For each training dataset, Ḋ^(ℱ)^_*m*_ of modality *m*, and frequency-band ℱ we identified candidate time-windows associated with correlation peaks in the respective CCTs (see section 4.2.1). In particular, we used 20.

#### 4.2.2. Correlated component maps

**Figure 1:**
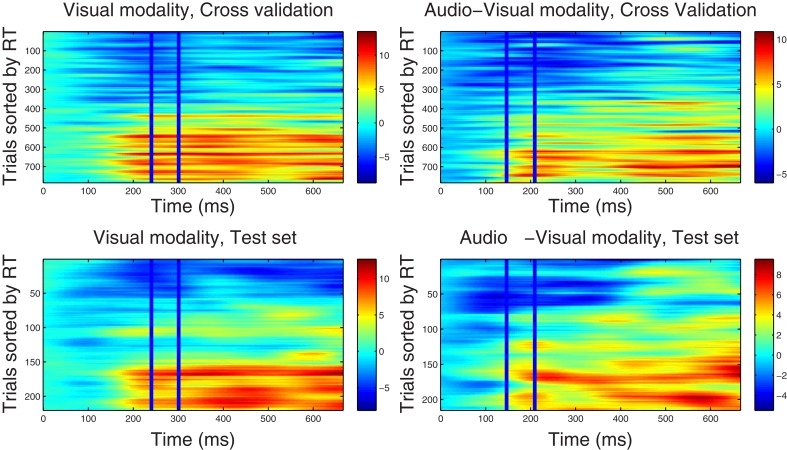
**Component correlation maps computed in the alpha band for the visual (*left column*) and audio-visual conditions (*right column*) obtained from the training dataset using a five-fold cross validation procedure (*top row*) and the test dataset (*bottom row*).** Trials are ranked by the reaction time achieved on each (trial number is listed on the vertical axes). Latency in ms after stimulus onset is shown on the horizontal axis. The blue vertical lines identify the time window where peak correlation coefficients were found. The *i*-th row in each graph represents the single-trial correlated component (SCC) of the *i*-th fastest trial. The amplitude of the SCC (spectral power) is represented on the color scale (red indicates maximum amplitude and blue minimum amplitude). Trials with faster reaction times have lower amplitude with in the optimal window, and as the reaction time increases amplitude of the extracted component increases.

**Figure 2:**
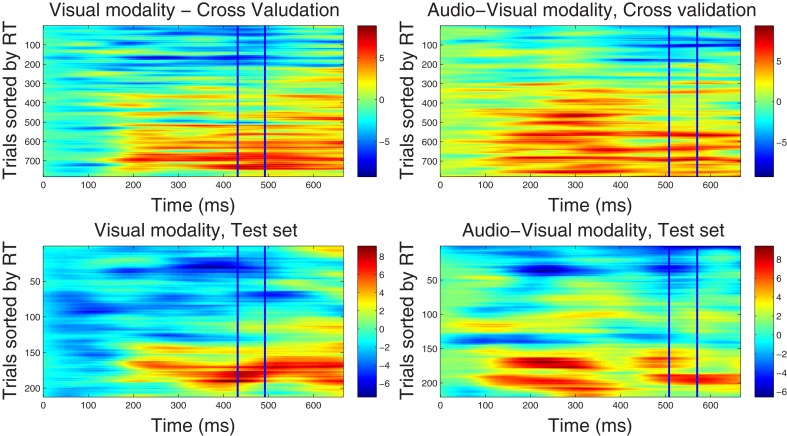
**Component correlation maps computed in the beta band (16–22 Hz) for the visual (*left column*) and audio-visual conditions (*right column*) obtained from the training dataset using a five-fold cross validation procedure (*top row*) and the test dataset (*bottom row*).** Trials are ranked by the reaction time achieved on each (trial number is listed on the vertical axes). Latency in ms after stimulus onset is shown on the horizontal axis. The blue vertical lines identify the time window where peak correlation coefficients were found. The *i*-th row in each graph represents the single-trial correlated component (SCC) of the *i*-th fastest trial. The amplitude of the SCC is represented on the color scale (red indicates maximum amplitude and blue minimum amplitude). Trials with faster reaction times have lower amplitude with in the optimal window, and as the reaction time increases amplitude of the extracted component increases.

In this step, the optimal spatial weighting vector **w**^ℱ^_*m*_ was estimated within the time window selected from each training dataset Ḋ^(ℱ)^_*m*_ using Equation (9). In order to characterize the temporal variation of each weighting vector, we calculated the correlated components for each trial and every time point by using Equation (10). Data from each trial was then sorted by reaction time and presented in Figures 1, 2 demonstrating how the strength (power) of correlated components varies across trials and epoch latency. Trials were sorted by the corresponding reaction time, so that “faster” trials are placed at the top row of each graph and trials associated with slower RTs are located at the lower rows. Graphs in Figures 1, 2 are referred to here as correlated components maps, adopting the naming convention used in discriminant component analysis. Such Maps were calculated for the *visual* and *audio-visual* modalities in the ℱ_α_ and ℱ_β 1_ frequency bands.

***4.2.2.1. Correlated component maps in ℱ_α_.*** Figure 1 shows CCMs for the visual (*left column*) and audio-visual (*right column*) conditions computed for the ℱ_α_ frequency-band datasets. The upper row displays the CCMs obtained from the training dataset Ḋ^(ℱ_α_)^_*m*_ using a five-fold cross validation procedure, and the lower row shows maps obtained from the independent test dataset ${\overset{..}{D}}_{m}^{\left({ℱ}_{\alpha }\right)}$. The time window used to estimate the spatial projection vector **w** is enclosed by vertical lines. Optimal windows ranged between 240 and 300 ms for the visual modality and between 150 and 210 ms in the audio-visual condition. Visual inspection of the upper-row graphs reveals that trials associated with faster RTs (lower rows in each graph) are accompanied by lower power in the alpha band within the corresponding optimal time windows, and vice versa. This pattern is present in the CCMs of both conditions. It is also evident that the duration of the correlated components (indicating the strength of the association between RT and spectral power) includes portions of the epoch outside of the optimal windows (i.e., between approximately 150–700 ms post-stimulus onset). This suggests that correlated components may not be strictly time-limited showing a broader latency span. As shown in the lower-panel CCMs, parameters derived from the Training dataset were successful in predicting the latency distribution of correlated components in a different set of trials.

***4.2.2.2. Correlated component maps in ℱ_β1_.*** The CCMs obtained from the dataset in ℱ_β 1_ frequency band are illustrated in Figure 2, showing optimal windows between 430–490 ms and 500–560 ms for the visual and audio-visual conditions, respectively. Similar to the CCMs obtained in the ℱ_α 1_ band, trials with faster responses are associated with lower amplitudes of their corresponding correlated components and vice versa. These results were closely replicated in the independent test set, providing further support to the capacity of our method to predict the correlated components in new data. Finally, we note that the duration of the correlated components extends beyond optimal windows (i.e., between approximately 150–700 ms post-stimulus onset). In the condition, in particular, correlated component strength showed a bimodal distribution across latency points, featuring an early peak around 250 ms and a later peak at approximately 500 ms after stimulus onset.

#### 4.2.3. Component correlation trace and forward model

**Figure 3:**
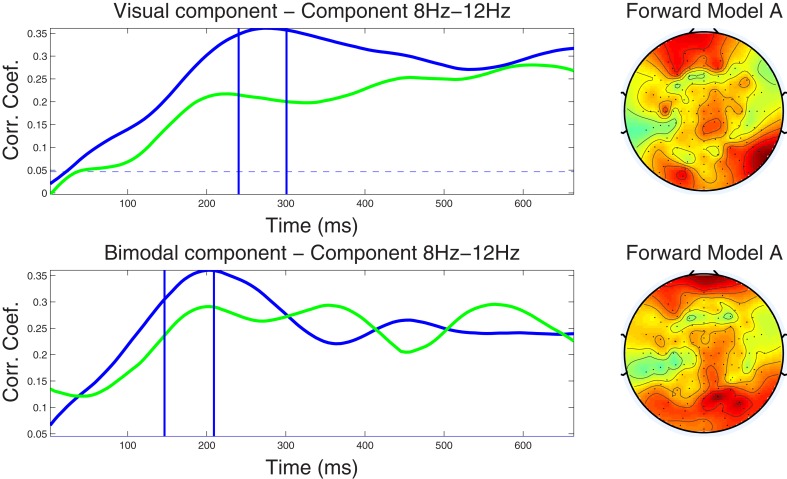
***Left column***: Component correlation trace (CCT) on the alpha-band (8–12 Hz) dataset for the visual (*top*) and audiovisual (*bottom*) conditions. The blue line shows the CCT from the training dataset, and the green line the CCT from the independent test dataset. The vertical blue lines identify the optimal window in which the correlated components were identified. The vertical axis shows the correlation coefficients between the extracted components and single-trial subject reaction times. ***Right column***: Forward model derived from the alpha-band dataset for the visual (*top*) and audiovisual (*bottom*) conditions. The hot to cold color scale indicates the average level of coupling between each correlated component and individual electrodes on the head surface.

**Figure 4:**
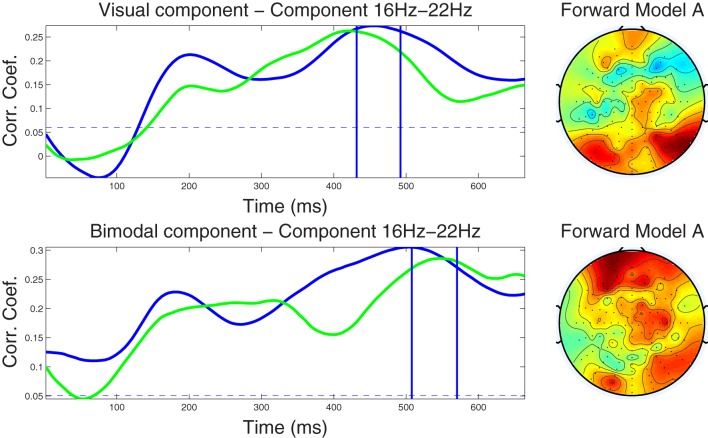
***Left column***: Component correlation trace (CCT) on the beta-band (16–22 Hz) dataset for the visual (*top*) and audiovisual (*bottom*) conditions. The blue line shows the CCT from the training dataset, and the green line the CCT from the independent test dataset. The vertical blue lines identify the optimal window in which the correlated components were identified. The vertical axis shows the correlation coefficients between the extracted components and single-trial subject reaction times. ***Right column***: Forward model derived from the beta-band dataset for the visual (*top*) and audiovisual (*bottom*) conditions. The hot to cold color scale indicates the average level of coupling between each correlated component and individual electrodes on the head surface.

As a means to summarize information present in the CCMs over trials, we calculated the CCT. The CCT shows the modulation of the correlation values of the optimal component across latency points. Figures 3, 4 shows the CCTs for the visual and audio-visual conditions obtained from the ℱ_α_ and ℱ_β_1__ frequency-band datasets, respectively. The correlation traces calculated from the corresponding training datasets are shown in blue; the green traces show the CCTs calculated in the independent test dataset. The right-hand panel of each figure demonstrates the scalp distribution of the corresponding forward model of the optimal spatial weighing vectors, calculated using Equation (12). These plots illustrate the scalp topography of the resulting components and the strength of the projection of the underlying neural source to each electrode.

***4.2.3.1. Alpha band dataset.*** Closer inspection of the upper left-hand panel in Figure 3, demonstrates a relatively slow rise of the CCT in the training dataset from the visual condition, from stimulus onset to approximately 270 ms (*r* = 0.36). A slightly earlier peak at approximately 220 ms (*r* = 0.22) was noted in the CCT obtained from the independent test dataset. Correlation values remained at relatively high levels for the remainder of the epoch (ranging between *r* = 0.27−0.31 in the training, and between *r* = 0.25−0.28 in the test dataset). The blue horizontal line in each graph indicates the significance level of *p* < 0.01 (FDR-corrected for multiple comparisons) for the null hypothesis that the two observations have no correlation on the component amplitudes with the optimal window. To calculate, we used a permutation test in which reaction times were first randomized across trials, then run the cross validation process and calculated the corresponding correlation index *r*. This was repeated for 1000 times providing a distribution of *r* values on which the null hypothesis was tested. The CCT for the audio-visual modality shows an earlier initial peak at around 200 ms for both the training and test datasets with peak correlation values of *r* = 0.36 and *r* = 0.29, respectively. After approximately 300 ms correlations level off ranging between *r* = 0.22 and *r* = 0.29 on both the training and test datasets. The similarity in shape (temporal pattern) across blue and green traces in both conditions further attests to the capacity of our method to predict the CCT pattern in new data. The forward model for the visual and audio-visual conditions on the ℱ_α_ band dataset is shown in Figure 3 (right column). Note that both plots, even though they were estimated independently for the two modalities, show similar topography, indicating maximum projection of the underlying neural source(s) to bilateral occipital and frontal electrodes, and to right parietal recording sites.

***4.2.3.2 Beta band dataset.*** The CCTs for the visual and audio-visual conditions derived from the ℱ_β 1_ band dataset are depicted in Figure 4. In the visual modality the CCT peaked at around 200 ms for both the training and test dataset with peak correlation values of *r* = 0.21 and *r* = 0.15, respectively. A second peak occurred at approximately 450 ms (*r* = 0.26) in the training and at 420 ms in the test dataset (*r* = 0.26). Very similar CCT waveforms were found for the audiovisual condition, each featuring an early peak at approximately 200 ms (*r* = 0.22 and *r* = 0.20, for the training and test datasets, respectively). The second peak in the CCT was found at 500 ms (*r* = 0.30) for the training and at 540 ms (*r* = 0.29) for the test dataset. Notably, the optimal latency window was estimated at a much later latency range (450–550 ms) as compared to the optimal windows established in the alpha band. Scalp-surface renderings of the forward model computed for the beta-band correlated components are shown in the fight-hand column of Figure 4, showing distinct similarities between conditions in overall topography.

We note that the peak components occurred between approximately 350 and 550 ms before the average response times in the corresponding conditions [mean RT for the visual condition was 780 ms (*SE* = 35) and 810 ms (*SE* = 29) for the audiovisual condition] which place them much before any motor activity and suggest that the extracted correlated components reflect activity from task related cognitive processes. Moreover, previous studies involving analyses of EEG and MEG data in similar tasks suggest that at these latencies neurophysiological events take place (in association cortices of the prefrontal and/or temporo/parietal regions) that support cognitive processes for the evaluation of stimulus properties in relation to existing memory representations.

#### 4.2.4. Generalizability of the method

As previously noted visual inspection of CCMs, CCTs, and corresponding forward model topographic maps suggest strong similarity between predicted values obtained from the cross validation and the actual values derived from the test dataset. A formal test of the degree of concordance between training- and test-data set derived CCT was performed by calculating the Pearson correlation between the two sets of values. The results are summarized in Table 1 demonstrating large coefficients for both conditions and frequency bands (ranging between *r* = 0.68 and *r* = 0.89).

**Table 1:** **Correlation coefficients between the amplitudes of CCT predicted by our method (obtained from the cross-validation dataset) and the amplitudes of CCT measured on an independent test dataset**.

**Dataset**	**Modality (m)**	**Optimal**	***r*-score**	***p*-value**
		**window (ms)**		
**D**^ℱ_α_^_*m*_	Visual	240–300	0.85	0.01× 10^−35^
**D**^ℱ_α_^_*m*_	Audio-visual	150–210	0.68	0.05× 10^−17^
**D**^ℱ_β1_^_*m*_	Visual	430–490	0.89	0.01× 10^−45^
**D**^ℱ_β1_^_*m*_	Audio-visual	510–560	0.78	0.01× 10^−26^

Optimal time-windows were identified in the alpha and beta1 frequency bands in the visual and audiovisual conditions.

#### 4.2.5. Comparison between visual and audiovisual conditions

**Figure 5:**
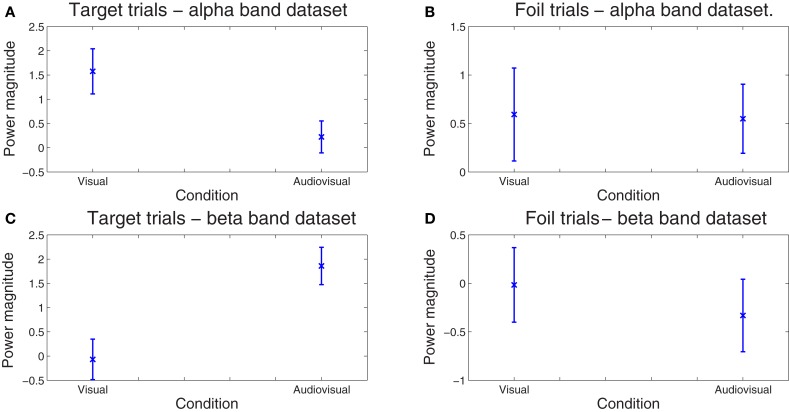
**Spread of correlated component amplitudes computed in the alpha (A and B) and beta bands (C and D) across trials for targets (A, C) and foils (B, D).** Whereas the difference in component amplitude was negligible between conditions on foil trials (*p* > 0.5), significantly higher values for the audiovisual as compared to the visual-only condition were found for targets in the alpha (*p* = 0.018) and beta bands ( *p* = 0.0007). No significant differences between conditions were found on foil trials alone (*p* > 0.9). Error bars correspond to the standard error.

Finally, the sensitivity of our method for deriving behaviorally-relevant features in single-trial EEG data that vary systematically with experimental manipulations was assessed on the amplitude (spectral power) of the derived correlated components. The dependent variable in these analyses was spectral power averaged across all time points comprising each optimal latency window for each trial. Mixed-models ANOVAs (SPSS mixed command) were employed in order to examine the effects of condition (visual, audiovisual), and stimulus type (target vs. foil) on component amplitude. Condition and stimulus type were treated as fixed factors while trial and subject as random factors. In the ℱ^α^ frequency-band dataset, there was a significant main effect of Condition, *F*_(1, 1585)_ = 5.165, *p* = 0.023. Inspection of the means in Figure 5 reveals significantly lower amplitude for the audiovisual condition [parameter estimate = 1.30, *SE* = 0.45, *t*_(1585.01)_ = 2.865, *p* = 0.004]. In the ℱ^β^ frequency-band dataset, the condition main effect, *F*_(1, 1584)_ = 5.07, *p* =0.024, was superceded by a condition by stimulus type interaction, *F*_(1, 1584)_ = 12.60, *p* =0.0001. Inspection of the means in Figure 5 reveals significantly lower amplitude for targets in the visual learning condition as compared to the audiovisual condition [parameter estimate = 2.35, *SE* = 0.66, *t*_(1584.01)_ = 3.549, *p* = 0.0001]. The two conditions did not differ on foils (*p* < 0.5). Notably the RT difference between conditions on foil trials also failed to reach significance (*p* > 0.5).

## 5. Conclusions

In this paper, we proposed a novel method for single-trial analysis of EEG signals in which we directly extract neural activity that maximally correlates to a continuous variable on interest. The method extents the applicability of single-trial discriminant analysis approach to paradigms for which the behavioral response is measured on a continuous variable (rather than a categorical variable). The method finds application to the problem of identifying correlates of quantifiable behavioral phenomena in measures of underlying neuronal activity. We demonstrated the effectiveness of this method in the analysis of EEG data obtained during performance of the recognition phase of a verbal learning task aiming to identify EEG correlates of the stimulus modality presentation effect. The method was successful in extracting two components, one in each of two frequency-bands, that significantly correlated with individual response times on a trial-by-trial basis. Further, the method permitted characterization of the temporal modulation of these components and their topographical coupling with recording electrodes by estimating the CCT of each component and its corresponding forward model. The ability of our method to predict the shape of the CCT in an independent data set was also established. Finally, the resulting components were shown to be sensitive to the stimulus modality effect establishing a neuronal correlate of the impact of presentation mode on learning and memory capacity.

## Conflict of interest statement

The authors declare that the research was conducted in the absence of any commercial or financial relationships that could be construed as a potential conflict of interest.
